# Effect of Auditory Cues on Placebo-Induced Hypoalgesia Using a Deactivated Transcutaneous Electrical Nerve Stimulation (TENS) Device: A Pilot Randomized Controlled Study in Healthy Volunteers

**DOI:** 10.7759/cureus.109314

**Published:** 2026-05-20

**Authors:** Giorgos Tzigkounakis, Spyridon Sotiropoulos

**Affiliations:** 1 Research, Health and Resilience Institute, Athens, GRC; 2 Physiotherapy, Aegean College, Athens, GRC; 3 Physiotherapy, University of Nicosia, Nicosia, CYP; 4 Physiotherapy, Musculoskeletal Physiotherapy Research Laboratory, University of West Attica (UNIWA), Athens, GRC

**Keywords:** auditory cue, hypoalgesia, pain modulation, placebo effect, pressure pain threshold, randomized controlled study, sham tens

## Abstract

Background

Placebo responses can be influenced by contextual and sensory cues. This pilot randomized controlled study investigated whether auditory cues modulate the analgesic response associated with a deactivated transcutaneous electrical nerve stimulation (TENS) intervention.

Methods

Thirty-eight healthy adults (aged 18-38) were randomly assigned to receive a deactivated TENS intervention with synchronized auditory cues or without sound. Participants were informed that the device was a modern, sensation-free TENS unit designed to relieve pain. Interventions were delivered in a single session under participant and assessor blinding. Pressure pain thresholds (PPT) were measured before and after the intervention using a handheld pressure algometer.

Results

The sound-enhanced group demonstrated a statistically significant increase in PPT following the intervention (p = 0.03), whereas no significant change was observed in the silent group. No significant baseline differences were identified between groups.

Conclusions

Auditory cues may augment the analgesic response within a sham TENS intervention in healthy individuals. These preliminary findings highlight the potential role of contextual sensory factors in modulating pain perception. However, given the pilot nature of this study and its retrospective trial registration, results should be interpreted with caution and require replication in larger, adequately powered trials before any generalizations can be drawn.

## Introduction

A placebo refers to a substance or intervention without a specific therapeutic effect for the treated condition, yet it may yield benefits due to the context in which it is administered and the expectations it generates. While definitions vary [1], placebo treatments lack active ingredients but may nonetheless influence clinical outcomes through psychological and contextual mechanisms [2].

Sensory cues, such as visual, tactile, or auditory signals, can modulate this neurobiological and psychological phenomenon, as contextual factors may shape patient perception and alter pain outcomes [3,4].

Transcutaneous electrical nerve stimulation (TENS) is frequently used as a non-pharmacological modality for pain management [5]. In research settings, deactivated or sham TENS devices are often employed as placebo controls to account for nonspecific effects [6,7]. However, even sham devices may emit sensory cues that may influence participant perception and outcomes [8,9].

While visual and tactile stimuli have been studied extensively in this context [10], the specific role of auditory signals, such as beeping sounds that simulate device activation, remains underexplored. Auditory cues may enhance perceived efficacy by mimicking active treatment.

This pilot randomized controlled study investigates whether an auditory signal augments the analgesic response of a sham TENS intervention compared to a silent sham device. Understanding such effects may refine sham controls and improve the design of pain interventions.

## Materials and methods

This was a participant- and assessor-blinded, parallel-group randomized controlled study to assess the effect of sound on the analgesic response within a sham intervention, conducted at Aegean Omiros College in Athens, Greece, between June 13 and 14, 2016. All outcomes were assessed immediately post-intervention, with no follow-up. No interim analyses or stopping guidelines were planned due to the pilot nature and short duration of the study. The study was approved by the Aegean Omiros College Ethics Committee and registered in ClinicalTrials.gov (Identifier: NCT06981260). All procedures complied with the Declaration of Helsinki. Written informed consent was obtained prior to enrolment, and participants were debriefed after study completion.

Participants

Inclusion criteria were: healthy adults (18-50 years) without acute/chronic pain or medication use (analgesic or centrally acting). Exclusion criteria included any orthopaedic, neurological (e.g., epilepsy), or other medical conditions, pregnancy, prior TENS experience, or participation in other studies. 

Thirty-eight adults (18-50 years) were recruited via online and on-campus advertising. Interested participants were invited to the lab, where they gave consent, following which they were screened and completed the protocol in a single session. No formal sample size calculation was performed, as this was a pilot study and no prior effect size estimates were available.

Randomization and allocation

**Figure 1:**
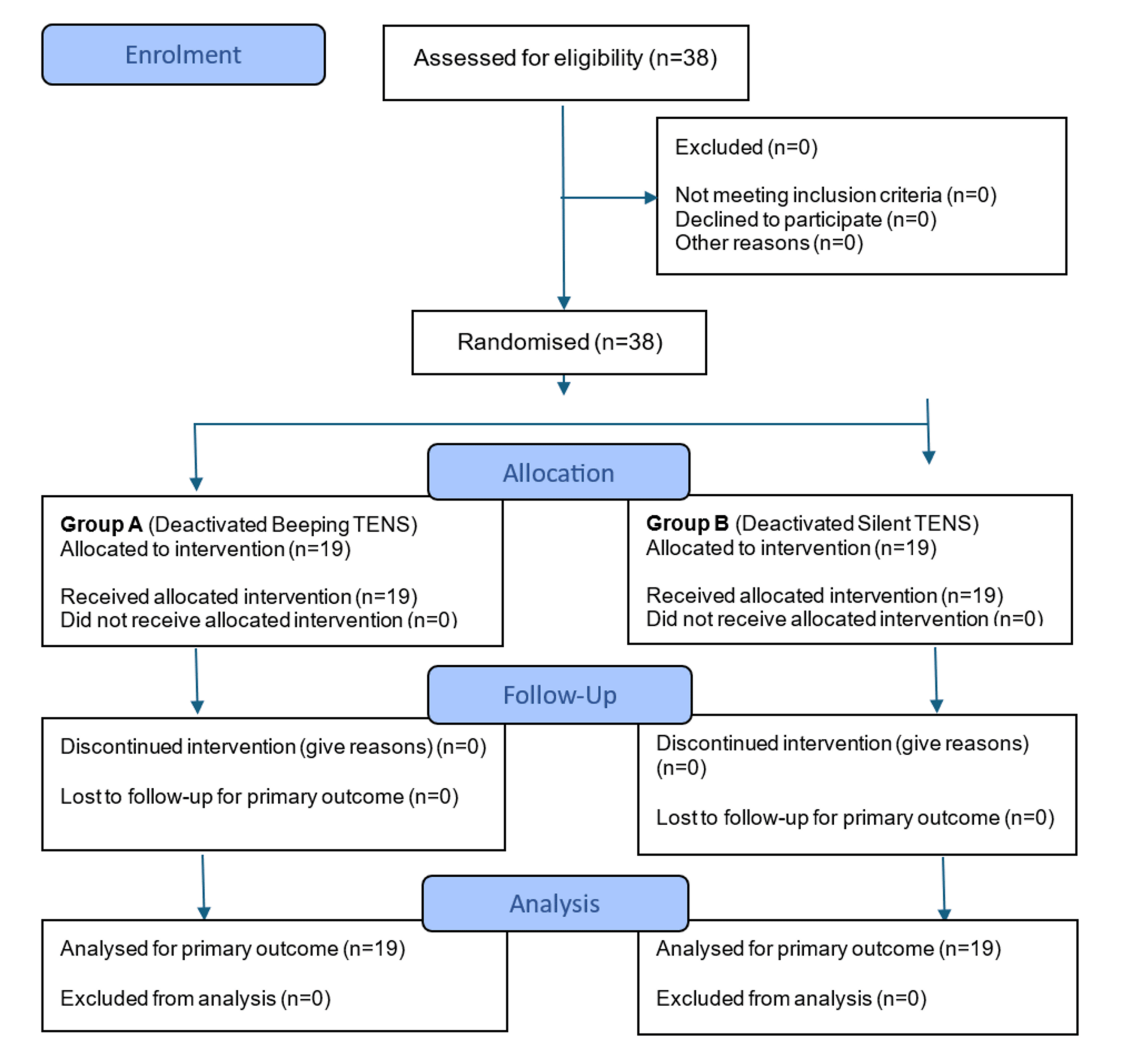
Flow diagram of participant progress through the study Group A: Participants using a deactivated TENS device with a repeated beeping sound; Group B: Participants using a silent deactivated TENS device. TENS: transcutaneous electrical nerve stimulation

Participants (n = 38) were randomly assigned 1:1 to one of two groups: (i) Group A (n = 19), using a deactivated TENS device with a repeated beeping sound, or (ii) Group B (n = 19), using a silent deactivated TENS device. This was done via computer-generated simple randomization, prepared by an independent researcher uninvolved in recruitment or assessment. Each participant was allocated sequentially according to the pre-generated list. The allocation sequence was securely stored and accessible only to the principal investigator. Allocation concealment was ensured by sealing the allocation sequence in a password-protected file, inaccessible to the recruiter and assessor until after participant enrolment and baseline assessment. Study documents and data were stored in password-protected electronic files on an institutional computer. Participant flow through the study is illustrated in Figure 1.

Blinding

Both participants and the independent assessor were blinded to group allocation and treatment modality. Participants were seated in a separate waiting area prior to their session and were therefore unable to hear or observe other participants' interventions, preventing cross-contamination of expectations between groups. The researcher who applied the interventions was not blinded, as he was responsible for delivering the assigned intervention. Blinding of the assessor was ensured by having the assessor leave the room during intervention administration.

Procedure

Upon arrival, all participants received standardized verbal suggestions to induce expectancy regarding hypoalgesia. Instructions described the intervention as a modern, pain- and sensation-free TENS-based device (see Appendices). However, as the researcher administering the intervention was not blinded to group allocation, the possibility of unintentional differences in demeanor between groups cannot be entirely excluded, representing a potential source of performance bias acknowledged in the Limitations section. 

Pressure pain threshold (PPT) was measured at baseline and immediately after the intervention. PPT was measured using a pressure algometer (Wagner Force Dial FDK/FDN Series; Wagner Instruments, Greenwich, Connecticut, United States) at the spinous process of the second lumbar vertebra (L2). Three measurements were taken at baseline and again immediately post intervention. The assessor, blinded to group allocation, recorded the values. Mean values from three trials were used for analysis.

The KWD-808I Multi-Purpose Health Device (Changzhou Yingdi Electronic Medical Device Co.,Ltd, Jiangsu, China) was used for all interventions and selected due to its availability on the premises at the time of the study. A dummy wire connected it to a laptop (MacBook Air, 11-inch; Apple Inc., Cupertino, California, United States), which played a beeping sound for Group A via the device's native speakers. The auditory stimulus was a 2680 Hz triangle waveform generated using Steinberg Cubase 8.0 (Steinberg Media Technologies GmbH, Hamburg, Germany), exported as an .mp3 file, and played on a 15-second repeat loop for the full five-minute intervention duration. The laptop was positioned at a fixed location relative to the participant throughout all sessions, and volume was maintained at a constant level across all participants. Participants saw the laptop-device connection but not the screen.

Participants in Group A received a five-minute application of deactivated TENS with a repeated beeping sound (interval 15 seconds, 2680 Hz triangle waveform), while Group B received a five-minute application of silent deactivated TENS. The frequency (2680 Hz) was selected to fall within the most sensitive range of human hearing (approximately 500-4000 Hz), where auditory sensitivity is highest due to auditory system tuning and ear canal resonance [11]. Electrode patches were placed identically on the lumbar area (L2) in all participants.

After the intervention, the assessor returned to re-measure PPT. Participants were asked at the end of the session whether they experienced any discomfort or adverse effects. No adverse events were reported.

Outcomes

The primary outcome was the change in PPT at L2 from baseline to immediately post-intervention. PPT was measured as the mean of three consecutive trials before and after the intervention.

Statistical analysis

Data were analyzed using IBM SPSS Statistics, version 23 (IBM Corp., Armonk, New York, United States). Mean PPT was derived from three measurements per time point. The primary analysis was a one-way ANOVA comparing post-intervention PPT between groups. To further evaluate treatment-related change, a supplementary analysis of between-group differences in change scores (post minus pre) was conducted using one-way ANOVA. Additionally, analysis of covariance (ANCOVA) was performed with post-intervention PPT as the outcome and baseline PPT as a covariate to account for within-subject correlation and adjust for any baseline differences. A secondary exploratory two-way ANOVA was conducted to examine the potential moderating effect of sex. A p-value < 0.05 was considered significant. As no missing data were observed (all 38 participants completed the study and were included in the analysis), no imputation or missing data handling procedures were required.

## Results

Thirty-eight participants were randomized (n = 19 per group; mean age = 27 years). Most participants were Greek, with 17/19 (89.5%) in Group A and 13/19 (68.4%) in Group B. All participants completed the study and were included in the final analysis. In Group A (deactivated TENS with sound), there were 14 male (74%) and five female (26%) participants. In Group B (silent deactivated TENS), there were 11 male (58%) and eight female (42%) participants. There were no significant differences in age or sex distribution between groups at baseline. Baseline demographic and ethnic characteristics of the participants are presented in Table 1.

**Table 1:** Baseline demographic and ethnic characteristics of participants by group Values are presented as n (%) for categorical variables and as mean for age (years). Group A: Deactivated TENS with sound; Group B: Silent deactivated TENS.

Characteristic	Group A (n = 19)	Group B (n = 19)
Sex		
Male	14 (74%)	11 (58%)
Female	5 (26%)	8 (42%)
Mean Age (years)	27	27
Ethnicity		
Australian	0 (0%)	1 (5.2%)
Albanian	0 (0%)	1 (5.2%)
Bangladeshi	1 (5.2%)	0 (0%)
Bulgarian	1 (5.2%)	1 (5.2%)
French	0 (0%)	1 (5.2%)
Greek	17 (89.5%)	13 (68.4%)
Russian	0 (0%)	2 (10.5%)

There was no statistically significant difference in pre-intervention PPT scores between the groups (F(1,36) = 0.42, p = 0.52). Following the interventions, a significant effect was observed in favour of the group that received deactivated TENS with sound compared to the silent deactivated TENS group (F(1,36) = 4.85, p = 0.03). Analysis of the influence of sex on the analgesic response showed no significant interaction (p = 0.105). No adverse events or side effects were reported by any participant during or after the interventions.

Outcomes

Comparison of mean PPT scores pre- and post-intervention showed an augmented analgesic response in Group A (sound; 45.09 to 49.68), whereas Group B (silent; 42.77 to 42.39) exhibited no such effect. Mean (SD) pressure pain threshold scores at baseline and post-intervention for each group are presented in Table 2.

**Table 2:** PPT scores (Newtons) at baseline and post-intervention for each group Group A: deactivated TENS with sound; Group B: silent deactivated TENS. PPT: pressure pain threshold; TENS: transcutaneous electrical nerve stimulation

	Group A (n = 19), mean (SD)	Group B (n = 19), mean (SD)	Total (N = 38), mean (SD)
Pre-treatment	45.09 (10.63)	42.77 (11.43)	43.93 (10.95)
Post-treatment	49.68 (9.46)	42.39 (10.92)	46.03 (10.73)

A one-way ANOVA was then conducted to compare the mean scores on the hypoalgesic effects of the two interventions between the two groups. In the pre-treatment scores, there was no statistically significant difference between the groups (F(1,36) = 0.42, p = 0.52). However, in the post-treatment scores, there was a statistically significant effect in favour of the intervention with sound (F(1,36) = 4.85, p = 0.03). Descriptive statistics and one-way ANOVA results for between-group comparisons at each time point are presented in Table 3.

**Table 3:** Descriptive statistics and ANOVA for PPT between groups F and p values are derived from one-way ANOVA (df = 1, 36) comparing groups at each time point. Statistical significance was set at p < 0.05. The standardized effect size for the post-intervention between-group difference was Cohen's d = 0.72. Group A: deactivated TENS with sound; Group B: silent deactivated TENS. PPT: pressure pain threshold; TENS: transcutaneous electrical nerve stimulation

Time point	Group A (n = 19), mean (SD)	Group B (n = 19), mean (SD)	F value	p value
Pre-treatment	45.09 (10.63)	42.77 (11.43)	0.42	0.52
Post-treatment	49.68 (9.46)	42.39 (10.92)	4.85	0.03

Mean (SD) PPT scores with 95% CIs are presented in Table 4. Baseline values did not differ significantly between groups. Post intervention, Group A (sound) showed a greater PPT increase than Group B (silent), indicating a group-level effect. The mean between-group difference in post-intervention PPT was 7.30 N (95% CI: 0.58 to 14.02), consistent with the statistically significant group effect observed in the primary analysis. To further assess treatment-related change, a one-way ANOVA on change scores (post minus pre) confirmed a statistically significant between-group difference (F(1,36) = 5.72, p = 0.022), with Group A demonstrating a mean increase of 4.60 N (SD 8.12) and Group B a mean change of -0.39 N (SD 4.07). Within-group paired t-tests showed a statistically significant pre-to-post improvement in Group A (t = -2.47, p = 0.024), while no significant change was observed in Group B (t = 0.41, p = 0.684). ANCOVA with baseline PPT as a covariate confirmed the group effect (p = 0.007, R² = 0.711). The standardized effect size was Cohen's d = 0.72 based on post-intervention scores and d = 0.78 based on change scores, both indicating a medium-to-large effect.

**Table 4:** Baseline and post-intervention PPT scores (Newtons) and 95% CIs for each group Group A: deactivated TENS with sound; Group B: silent deactivated TENS. PPT: pressure pain threshold; TENS: transcutaneous electrical nerve stimulation

Timepoint	Group A (n = 19), mean±SD (95% CI)	Group B (n = 19), mean±SD (95% CI)	Total (n = 38), mean±SD (95% CI)
Pre-treatment	45.09±10.63 (39.96, 50.21)	42.77±11.43 (37.26, 48.281)	43.93±10.95 (40.33, 47.53)
Post-treatment	49.68±9.46 (45.13, 54.24)	42.39±10.92 (37.12, 47.65)	46.03±10.73 (42.51, 49.56)

Although not a part of the initial hypothesis, the potential influence of sex on the placebo response was also investigated using ANOVA. The analysis showed that sex was not a statistically significant factor (F(1,34) = 2.41, p = 0.130), suggesting that the placebo effect may not be sex dependent. Two-way ANOVA results for the effects of group and sex on post-treatment PPT are presented in Table 5. It should be noted that the two-way ANOVA partitions variance differently from the primary one-way ANOVA; the modest attenuation of the group effect (from p = 0.03 to p = 0.053) when sex is included in the model reflects the redistribution of variance rather than a contradiction of the primary finding. Partial eta-squared (η²) for the group effect in the two-way ANOVA was η² = 0.106.

**Table 5:** Two-way ANOVA results for the effect of group and sex on post-treatment PPT scores F-statistics from two-way ANOVA are reported as F(df1, df2). Statistical significance was set at p < 0.05. Partial eta-squared (η²) reflects the proportion of variance explained by each effect. * deactivated TENS with sound vs. silent deactivated TENS; **male vs. female. PPT: pressure pain threshold; TENS: transcutaneous electrical nerve stimulation

Effect	F value	p value	partial η²
Group*	F(1,34) = 4.03	0.053	0.106
Sex**	F(1,34) = 2.41	0.130	0.066
Group × Sex	F(1,34) = 2.77	0.105	0.075

## Discussion

In recent decades, there has been a notable shift in the way science, psychologists, and clinicians perceive the placebo effect [12]. Accumulating evidence demonstrates that placebos can elicit specific physiological reactions, including measurable changes in brain chemistry [13] and circuitry, as well as distinct neurobiological underpinnings [14].

As a result, many scholars argue that placebos may have clinical significance beyond their use in double-blind experimental trials [12] as they can represent a promising model that could allow us to shed new light on mind-body interactions [15]. Moreover, since the mechanisms that are activated by placebos are the same as those activated by drugs, which suggests a cognitive/affective interference with the intervention's actions [13], they can be considered as potential tools that can assist in enabling and/or enhancing a therapeutic process. Thus, identifying factors and elements that can contribute or assist in initiating, enabling, and/or enhancing placebo effects can be of great significance and value for science.

This pilot randomized controlled study evaluated whether an auditory cue modifies pain perception during a sham TENS intervention in healthy volunteers. The results provide preliminary support for this hypothesis, though caution in interpretation is warranted given the pilot nature of the study and the small sample size (n = 38), resulting in limited statistical power and wide confidence intervals.

Analysis of the PPT revealed that, while both groups were comparable at baseline, Group A (deactivated TENS with sound) exhibited a greater increase in PPT post intervention compared to Group B (silent deactivated TENS). 

Both groups received identical verbal instructions to standardize expectations, consistent with the expectancy theory, which posits that verbal suggestions can potentiate placebo responses. To minimize potential bias, participants with prior experience with TENS or similar devices were excluded from the study. Randomization was employed to ensure equal allocation. An exploratory analysis considered sex as a potential moderating factor. This two-way ANOVA was exploratory and secondary to the primary one-way comparison; the attenuation of the group effect observed when sex is included in the model should be interpreted accordingly. The results of the two-way ANOVA indicated that neither sex nor the interaction between sex and group assignment significantly affected the placebo response, further supporting the findings. The use of sound in this context may act as a subtle psychosocial signal that enhances the plausibility of treatment.

In conditioning and expectation-based placebo research, neutral sensory cues, including auditory stimuli, can acquire therapeutic meaning when repeatedly paired with active treatment, eventually shaping expectancy and enhancing placebo responses [16]. However, in the present study, participants with prior experience of TENS were excluded to avoid confounding effects. Thus, classical conditioning via personal prior exposure was unlikely. Instead, the observed augmentation of the analgesic response in the auditory cue group may be attributed to symbolic associations and expectancy mechanisms. The beeping sound, mimicking the operational feedback of a functioning medical device, likely reinforced participants’ perception of treatment authenticity, reinforcing expectations that can act as powerful modulators of treatment responses [17]. Such expectancy-driven modulation of pain aligns with established cognitive models of placebo analgesia, in which contextual cues act as top-down modulators of sensory processing [10]. Interestingly, Group B (placebo without sound) did not demonstrate a significant placebo response, despite the standardized verbal cues. It is possible that, in healthy volunteers not experiencing pain or a desire for therapeutic relief, the absence of additional sensory cues, such as sound, limited the effectiveness of the verbal suggestion alone.

The methodology employed, measurement of PPT via a pressure algometer at the lumbar spine, was chosen to avoid confounding by prior pain experience. While it could be argued that repeated pressure application might resemble a manual therapy mobilization, the short, non-oscillatory application in this study does not replicate the duration or technique of spinal mobilization procedures, which require cycles of 60 seconds, frequency of 0.5Hz - 2Hz [18-21] in pre-set intervals and sets, and thus, is unlikely to have produced hypoalgesia independently.

In addition to the main study aims, an exploratory analysis to assess sex differences in the placebo response was conducted. While recent work in chronic pain populations has shown stronger placebo effects in women, largely mediated by conditioning and reinforced expectations [22], broader meta-analyses report limited or inconsistent sex effects in clinical trials [23]. More broadly, placebo responses are shaped by a complex interaction of expectancy, conditioning, and contextual cues, with emerging evidence supporting their measurable neurobiological and clinical relevance across settings [24]. In our sample, neither sex nor the group × sex interaction significantly influenced post-intervention PPT scores. Thus, within our healthy volunteer context, the analgesic response did not appear sex-dependent. Given these findings, along with the modest sample size typical of pilot studies and the inconsistent evidence in the literature, future research with larger, adequately powered samples is needed to clarify whether, and under what conditions, sex moderates the placebo analgesic response.

Limitations

The primary limitation of this pilot trial is the small sample size (n=38), which may introduce standard error and limit the precision of effect estimates. Nevertheless, the modest sample allowed for efficient recruitment, randomization, and implementation, enabling timely exploration of the research question. External validity is also limited. Although the study aimed to recruit participants from varied ethnic and socioeconomic backgrounds, this was not achieved. The age range was narrow (18-38 years), further restricting generalizability to broader populations, including minors and older adults. Additionally, there was a notable imbalance in sex distribution between groups (Group A: 74% male; Group B: 58% male), which, despite a non-significant group × sex interaction, may have influenced the magnitude of the placebo response, given the modest sample size. Moreover, the absence of post-intervention follow-up precludes conclusions about the durability of placebo effects. In parallel, this study did not include a no-treatment or waiting-list control group, which limits the ability to distinguish a true placebo effect from natural history or regression to the mean. Accordingly, the findings should be interpreted as evidence that auditory cues may augment the response to a sham intervention, rather than as evidence of placebo analgesia generated from a zero baseline.

Furthermore, blinding integrity was not formally assessed. While physical separation of waiting areas prevented cross-contamination between participants, the absence of sound in Group B may have led individual participants to question device activation despite identical verbal suggestions. Future studies should incorporate credibility measures such as the Credibility/Expectancy Questionnaire (CEQ). Additionally, while verbal suggestions were standardized via a fixed script, the unblinded researcher introduces a potential risk of investigator bias through subtle, unintentional differences in behavior between groups. Beyond this, the study did not examine the potential influence of different sound frequencies or waveforms on outcomes. While the chosen auditory stimulus fell within the most sensitive human hearing range, its effects may not generalize to other acoustic characteristics. Future studies should explore whether different auditory profiles elicit distinct neurobiological or behavioral responses.

## Conclusions

The clinical implications of these findings are twofold. First, contextual elements such as auditory cues may represent a simple, ethical, and non-invasive means to augment the effectiveness of therapeutic modalities, especially when combined with evidence-based intervention. Second, clinicians should be mindful of the ethical considerations surrounding the use of placebo elements. While leveraging contextual cues as adjuncts to active treatments may enhance outcomes, exclusive reliance on inert interventions is neither ethical nor recommended, as it may undermine trust if discovered by the patient.

This pilot study provides preliminary evidence that the inclusion of an auditory cue, specifically a device-generated sound, may augment the analgesic response within a sham TENS intervention in healthy volunteers. These findings are preliminary, primarily applicable to healthy volunteers, and should not be extrapolated to clinical practice without caution. Larger, adequately powered, prospectively registered clinical studies are essential to confirm these observations before any practical or therapeutic application can be considered.

## Appendices

Verbal suggestions/information given to the subjects

> This research investigates the immediate hypoalgesic effects that the application of a modern, cutting-edge, absolutely pain and sensation-free device, identical to the traditional TENS device, can provide. > It is a cutting-edge technology that became available to our university some months ago. > It has been tested extensively in various European countries; however, in Greece, it is something new. This is why we seek evidence from trials conducted within Greece to support its cutting-edge use and effectiveness. > An expensive software alters the TENS frequencies in such a manner that minimises the sensation to the patient and at the same time provides advanced analgesic relief.  > Most patients feel absolutely nothing at all; however, there is a small percentage of patients who feel a small tingling similar to all TENS devices. > Nevertheless, the analgesic and hypoalgesic effects that this machine provides are immediate, and as such, it is believed to conquer the sports rehabilitation equipment and become a necessity in all modern physiotherapy clinics.
